# Supplementary material for: Polyketide synthase gene clusters in the ericoid mycorrhizal fungus Oidiodendron maius: genome-wide prediction, expression profiling, and functional characterization of knockout mutants
Source: Front Plant Sci. 2026 Sep 16;17:1924430. doi: 10.3389/fpls.2026.1924430 (PMC13623903; doi:10.3389/fpls.2026.1924430)
Supplement: Supplementary file 1 [file SupplementaryFile1.zip › Supplementary Materials/Supplementary Folder 1.zip!/Oidma1_AssemblyScaffolds_Repeatmasked/knownclusterblast/cluster38/jgi.p_Oidma1_59034_mibig_hits.html]

| MIBiG Protein | Description | MIBiG Cluster | MiBiG Product | % ID | % Coverage | BLAST Score | E-value |
| --- | --- | --- | --- | --- | --- | --- | --- |
| CAL80830.1 | dehydrogenase-related\_protein | BGC0000997 | Glidobactin\_biosynthetic\_gene\_cluster | 30 | 51.5544041451 | 96 | 7.6e-20 |
| CAG44632.1 | SriL03.9 | BGC0000712 | Paromomycin\_biosynthetic\_gene\_cluster | 32 | 51.8134715026 | 95 | 1.3e-19 |
